# An Improved Forest Fire Detection Method Based on the Detectron2 Model and a Deep Learning Approach

**DOI:** 10.3390/s23031512

**Published:** 2023-01-29

**Authors:** Akmalbek Bobomirzaevich Abdusalomov, Bappy MD Siful Islam, Rashid Nasimov, Mukhriddin Mukhiddinov, Taeg Keun Whangbo

**Affiliations:** 1 Department of Computer Engineering, Gachon University, Sujeong-gu, Seongnam-si 461-701, Gyeonggi-do, Republic of Korea; 2 Department of Artificial Intelligence, Tashkent State University of Economics, Tashkent 100066, Uzbekistan

**Keywords:** forest fire, fire detection, Detectron2, deep learning, fire image dataset

## Abstract

With an increase in both global warming and the human population, forest fires have become a major global concern. This can lead to climatic shifts and the greenhouse effect, among other adverse outcomes. Surprisingly, human activities have caused a disproportionate number of forest fires. Fast detection with high accuracy is the key to controlling this unexpected event. To address this, we proposed an improved forest fire detection method to classify fires based on a new version of the Detectron2 platform (a ground-up rewrite of the Detectron library) using deep learning approaches. Furthermore, a custom dataset was created and labeled for the training model, and it achieved higher precision than the other models. This robust result was achieved by improving the Detectron2 model in various experimental scenarios with a custom dataset and 5200 images. The proposed model can detect small fires over long distances during the day and night. The advantage of using the Detectron2 algorithm is its long-distance detection of the object of interest. The experimental results proved that the proposed forest fire detection method successfully detected fires with an improved precision of 99.3%.

## 1. Introduction

Forest fires, also known as wildfires, are one of the most devastating events that have occurred in recent years, causing loss of life and damage to property. Between 2002 and 2016, an estimated 4,225,000 km^2^ of land was burned due to uncontrollable fires [1]. Forest fires can be classified into two main categories: natural and human-caused. Dry weather, wind, lightning, volcanoes, meteors, coal-seam fires, heating, and smoking are examples of natural causes, while cooking, accidental, or intentional acts of negligence are examples of human-created fires. Both natural and human-created fires significantly affect wildlife and human life. Early detection of fire can be key to preventing this kind of unexpected event and can save many lives and resources. In 2022, a wildfire reported in Hapcheon County (approximately 35 km southwest of Daegu city, southeastern South Korea) burned an area of approximately 675 hectares, and approximately 460 residents from Hapcheon and Goryeong counties were evacuated. Human activity accounts for 90% of all wildfires, and lightning is the highest among the remaining 10% of fires [2]. Wildfire toxic gases affect tropospheric ozone levels, which in turn affect humans and wildlife [3].

Fast detection is key to reducing the overall effect. Traditional human surveillance is expensive and not as efficient as a detection model [4]. The management of humans and the maintenance of resources are time-consuming and costly. Automation is a much better and more accurate approach. Weather conditions, temperature, rain, and wind can affect fire detection. Therefore, collecting data in real time is much better with a lower cost [5].

Object detection began with the machine-learning concept, which was first introduced in 1986. However, owing to technological limitations, the machine-learning approach did not contribute significantly. In late 2000, deep learning was introduced, leading to a faster and more accurate deep learning object detection algorithm. Compared to other deep learning algorithms, such as Mask R-CNN and Faster R-CNN, the efficiency, robustness, and accuracy of Detectron2 for object identification increased significantly in 2017. Detection systems can be divided into three main categories: wireless, satellite, and large-area monitoring. The sensor has almost the same approach, but its range is limited [6], with a shorter lifetime.

Detectron2 is a robust, reliable, and automatic fire detection approach using a mask-RCNN. Detecting fire is challenging because of the size, color, motion, speed, approach, sunlight, and a combination of these different factors. Although these factors make fire detection challenging, the use of the dataset, training model, and data angle can achieve maximum accuracy.

The major contributions of the proposed method are as follows:

(1) An automated forest fire detection method was developed to reduce natural disasters and forest resource loss.
(2) To train the proposed model, we collected a large custom dataset with two classes, fire, and non-fire, with different scenarios (day and night) of fire and flame, light, and shadows. The dataset is available on GitHub for public use. We used the LabelMe data annotation tool, which annotates fires and non-fires using a polygon instead of a rectangle.
(3) The forest fire detection accuracy was improved using fire and non-fire images and data augmentation techniques. In addition, the proposed model significantly increases the precision and decreases the false detection rate, even in small fire regions.

The rest of the study is structured as follows: Section 2 reviews the literature on traditional and deep learning methods used to identify particular fire regions. The proposed fire detection method is described in detail in Section 3. In Section 4, we discuss the experimental findings derived from quantitative and qualitative experiments and our dataset. Some of the limitations of the proposed approach are discussed in Section 5. The paper concludes with a summary of our results and directions for further research in Section 6.

## 2. Related Work

Forest fire detection technologies can be divided into two main categories: machine learning, deep learning, and computer vision methods and the use of sensor-based methods. The sensor-based method is limited to some extent. To overcome these limitations, we designed and developed a deep learning method (Detectron2) for object detection, with additional information requirements on location and shape [7]. The most common approaches to detect objects in deep learning are image-based convolutional neural networks (CNNs) [8,9,10,11], fully convolutional networks [12], cost-effective deep CNN architecture for fire detection from video [13], and faster R-CNNs [14]. In recent studies, it was observed that object-based detection in the industry had gained popularity over deep learning [15,16].

### 2.1. Forest Fire Detection Using Machine Learning and Deep Learning Approaches

Toulouse et al. [17] developed a new method to detect the geometrical characteristics of a fire depending on its position, surface, and length. In this study, the fire color was categorized into pixels. Moreover, the pixels were classified based on the average intensity of the non-refractory images. Jian et al. [18] introduced an upgraded boundary detection operator, and their model used a multistep operation. However, the abstraction of the model was only applied to simple and stable fire and flame images. Researchers worldwide have used a new algorithm based on fast Fourier transform (FFT) to detect fires. Turgay [19] developed a real-time fire detector that combined background and foreground color frames. However, the real-time color-based program does not provide a better output because of the smoke and shadow. In [20], based on the dynamic textures of smoke and flame, fire was detected using dynamic systems (LDS).

Recently, deep-learning-based object detection has become more popular than sensor-based object detection. In [21], Park et al. proposed the ELSTIC-YOLOv3 model to detect small objects, and in the same study, they mentioned the dynamic fire tube, a characteristic of fire. The research team in [22] proposed a CNN-based model with an average precision accuracy of 83.7%. Furthermore, in [23,24,25,26], an approach to improve fire detection technology was presented. In CNN, the challenge is to achieve high accuracy by training with a large dataset, which is an expensive process. Recent studies show fire detection systems have changed from traditional approaches to object-based detection systems [14], which have been rising in popularity in the industry.

### 2.2. Forest Fire Detection Based on YOLO, Transformers, and Detectron Approaches

The use of object-based detection algorithms has been recently reviewed, from initial algorithms, stemming from the viola-Jones detectors, from the main research line that can be separated into two groups based on the number of stages. Single-stage detectors are associated with only one look algorithm. YOLO version (v2, v3, v4 and v5) and the single shot multi-box Detector (SSD) are the best examples of single-stage detection [27,28,29,30]. This type of detector has some limitations. The large class imbalance between foreground and background boxes affects the prediction accuracy. The main features of single-stage detectors are the detection of the boundary boxes and the object classification done by the same single feed-forward fully convolution network. On the Detectron2 platform, a deep learning object identification model for detecting forest fires and accompanying smoke plumes was implemented [31].

Transformers were proposed to eliminate the limitation and to model the long-range interactions between input patches using a self-attention mechanism, which is at the core of transformers. Transformers showed good performance when applied in computer vision tasks such as video processing [32], image super-resolution [33], object detection [34] and segmentation [35], and image classification [36], i.e., Vision Transformer (ViT) [37], DeiT (Data-efficient Image Transformers) [38], and Medical Transformer (MedT) [39]. Researchers presented the first study in [40], which investigated the possibility of using vision transformers in the context of forest fire segmentation. TransUNet and MedT, two vision-based transformers, were used. Two frameworks were created based on the previous picture transformers that were tailored to their complicated, non-structured environment, which they tested using different backbones with optimization for forest fire segmentation. Self-attention has three advantages for effectively detecting fire pixels: There are fewer parameters. The model’s complexity is reduced, as are the number of parameters. As a result, the computational power required is even lower, and the pace is faster. Because each phase of the attention mechanism is independent of the preceding step’s calculation results, it can be processed in parallel similar to a CNN, with good results. Close attention must be paid to the crucial points. Even if the text or visual material is somewhat long, the vital points can be grasped from the center without losing important information. In general, limited attention can be focused on crucial information, saving resources and receiving the most useful information as rapidly as possible [41].

Two-stage detectors originate from the Region-Based Convolutional Neural Network (R-CNN) family [42]. Two-stage detectors follow the initial single-stage detector stage of a compilation of bounding boxes succeeded by the feature extraction method and then the final stage based on extracted features. This feature is sometimes slow, which prompted the development of a modified accelerated first step, the so-called Fast R-CNN model that is used in pretrained image classification backbone models such as ResNet for a faster approach [43].

In terms of fire detection, remote and close image sensing systems apply CNNs for object detection tasks. The majority of the previous image processing development was tailored to specific sets of images, as designing an algorithm could achieve high specificity. However, due to the important use of datasets, organization necessity is still the greatest challenge. In [7], Guede-Fernández proposed a Detectron2-based object detection system for forest fire detection. The model detects forest fires with quite high accuracy, but for close fire detection, the accuracy is not up to the mark. Moreover, at night and on a cloudy day, the model shows its own limitations. In our proposed model, we upgrade forest fire detection by using Detectron2 with high accuracy to overcome these limitations.

## 3. Proposed Forest Fire Detection

### 3.1. Forest Fire Dataset

In object detection, the main limitation is the collection of data for implementation in a custom training model. To address this problem, we collected forest fire data from different databases and used several computer vision techniques to enhance the dataset. To achieve more accurate results, we created two classes of datasets: fire and non-fire. The dataset was publicly available, and some images were collected from Google. To train our dataset first, we resized all images at the same height and width to avoid unexpected results or errors. After data collection, the dataset was small. To increase the dataset, we searched the Internet for videos of forest fires and captured frames of those videos. Our training dataset was compressed with 5200 day and night forest fire images and non-fire images to differentiate fire images from non-fire images to achieve maximum accuracy. Small datasets prevented us from achieving the desired accuracy, as shown in Table 1. Consequently, we employed data-augmentation techniques to expand the dataset. The following section describes the collection and expansion of the custom dataset in detail.

**Scheme 1:**
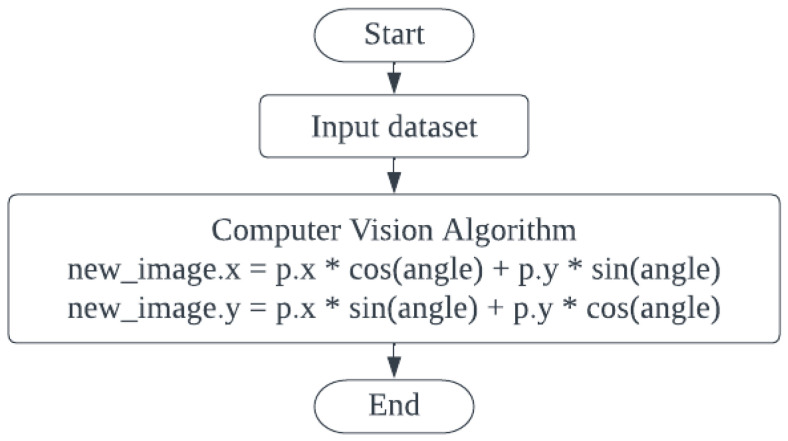
Image processing (rotation).

**Figure 1:**
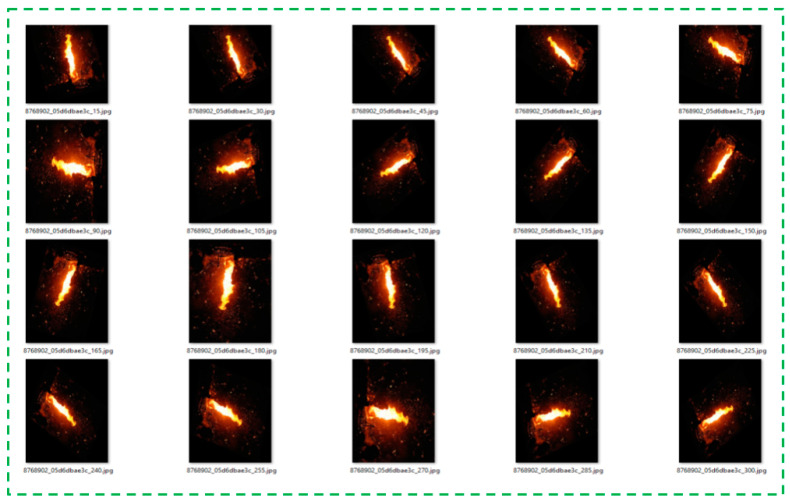
Example images of forest fire rotation from different angles.

We increased our dataset using a computer vision algorithm to rotate each image at 15° angles to 360°, as shown in Figure 1. Our dataset increased by 23 times by applying this technique. As mentioned earlier, we compressed 5200 images in our dataset. After augmentation, the total number of images was extended to 119,600, and we had 10,120 fire-like images to prevent false-positive results, as presented in Table 2, and Scheme 1 shows the flow chart. The simple linear algebra will provide the equations to rotate any point p and q with an angle. Detectron2 provides good results on a small dataset.

**Figure 2:**
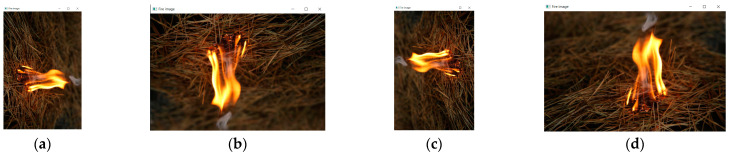
Example images of forest fire rotation from different angles. (**a**) 90° rotation; (**b**) 180° rotation, and (**c**) 270° rotation; (**d**) is the original image.

However, with a large dataset, the fire detection accuracy showed improved results compared with the small dataset. Therefore, it was preferable to extend the training dataset. Second, we rotated all forest fire images to 90°, 180°, and 270° (Figure 2). When image rotation values are greater than 15°, the output is almost similar, whereas when image rotation is approximately 90°, we lose our forest fire image’s region of interest.

**Figure 3:**
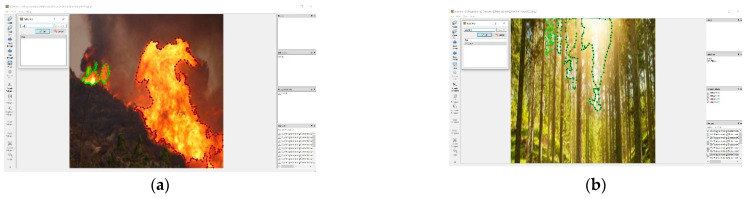
Image annotation; (**a**) fire, (**b**) non-fire.

We used LabelMe software to annotate our images, which is an important step in the training process for Detectron2 as shown in Figure 3. Our level file was a JSON file that was saved in the same folder as the training file. In addition, in Detectron2, all image sizes must have the exact size (height and width). Therefore, before annotating the images, we resized all images to the same height and width using OpenCV. Furthermore, we added non-fire images to our training set and labeled them as such. The purpose of training non-fire images was to reduce the number of false detections.

In our dataset, each image was rotated by 15° to 360°, resulting in 23 images from the same image. If the images are labeled manually, we lose considerable time in performing the same task repeatedly. Hence, we used the affine transformation method to rotate the same image. Image transformation was presented in a matrix using NumPy [9].

### 3.2. System Overview

**Scheme 2:**
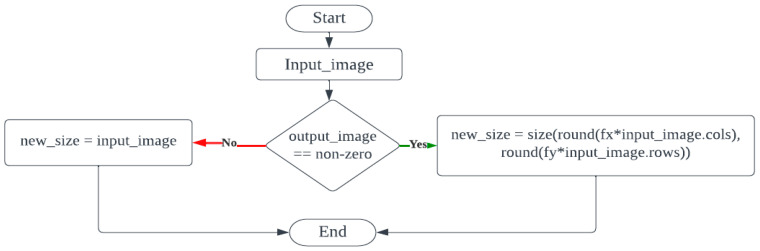
Image resizing.

**Figure 4:**
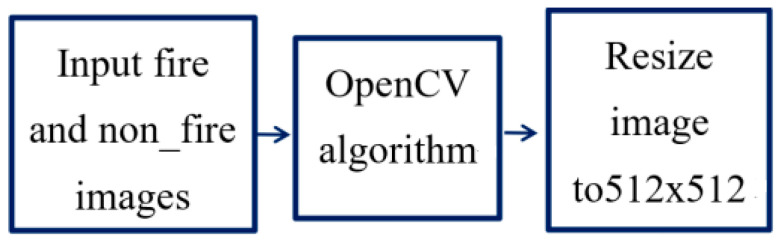
The overall process of resizing images.

In this subsection, we propose a method to detect fires more accurately and quickly. We resized and shaped the forest fire images. Several techniques were applied to develop the dataset. First, we resized the input images to 224 × 224, 320 × 320, and 512 × 512 using OpenCV2, as shown in Figure 4. In our study, we used 416 × 416 images to increase the accuracy and reduce the false detection rate of our forest fire model. Before training our model in the CNN, we implemented data augmentation and image contrast information processing. In Scheme 2, the flow chart of image resizing is shown; i.e., output_image. It has the size new_size (when it is non-zero) or the size computed from input_image.size(), fx, and fy.

### 3.3. Forest Fire Detection

In recent years, Detectron2 has been used to detect both moving and static objects in commercial research. Detectron2 has better accuracy compared to other object detection libraries or frameworks. Detectron2 is implemented in PyTorch and Cuda, providing a robust, fast, and more accurate result. As mentioned earlier, we used 5200 forest fire images. Real-time object detection using Detectron2 was faster and more accurate. Detectron2 uses a deep-learning approach to detect objects. PyTorch (1.13.0) and Cuda (11.7.0) were used to verify the accuracy of the model-tested images. We used the default algorithm without any change in the training model, and the results after 50,000 iterations are presented in Table 3. Furthermore, a default image hue of 0.1, saturation of 1.5, and exposure of 1.5 were used.

**Figure 5:**
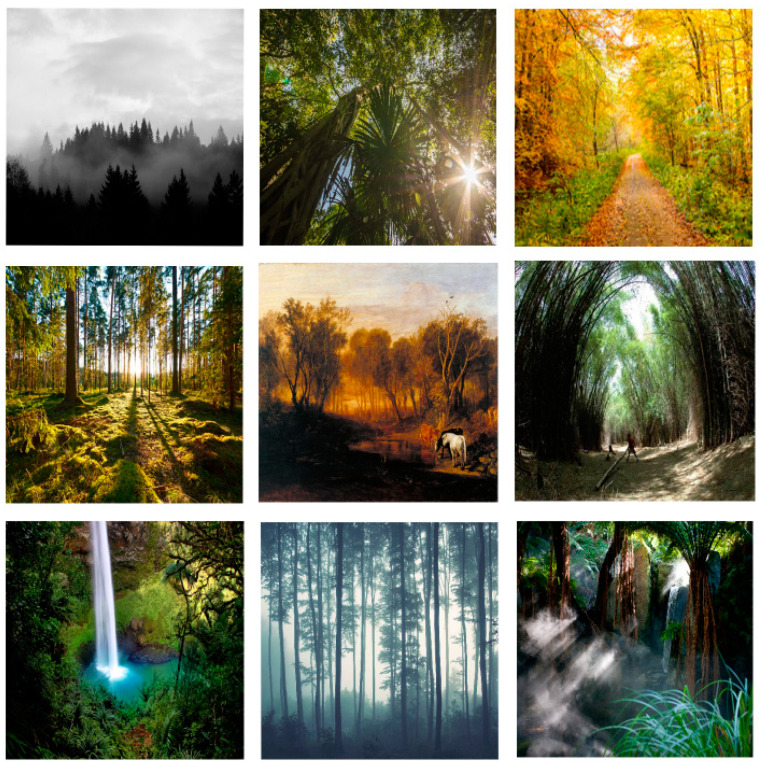
Forest fire-like lights (non-fire) [6].

In Detectron2, we set the input images of forest fire and non-fire to 512 × 512 in the same manner. As shown in Table 3, the results were obtained for the training and testing accuracies with different indicators. Mask_rcnn_50_FPN_3x had a high training accuracy of 83.8% and 79.8% in 62 h. The following results were obtained: Keypoint_rcnn_R_50_FPN_3x, 82.4%, and testing accuracy of 77.8%. The accuracy and testing of Mask_rcnn_50_FPN_3x and Keypoint_rcnn_R_50_FPN_3x were similar. However, the difference was in the model training time with a small weight. Increasing accuracy requires more training time, which is costly. The challenge of training in Detectron2 is to find PyTorch’s capability with Cuda in the GPU mode. Human eyes can easily differentiate forest fire images from non-fire images based on the color of the fire, size, shape, and reflection [5]. Unlike human eyes, our model can differentiate between non-fire and fire images owing to the shape, color, and similar environment, which can lead to false detection. Therefore, a large dataset leads to more accurate object detection. Figure 5 shows forest fire-like lights images such as sun, haze and others.

**Figure 6:**
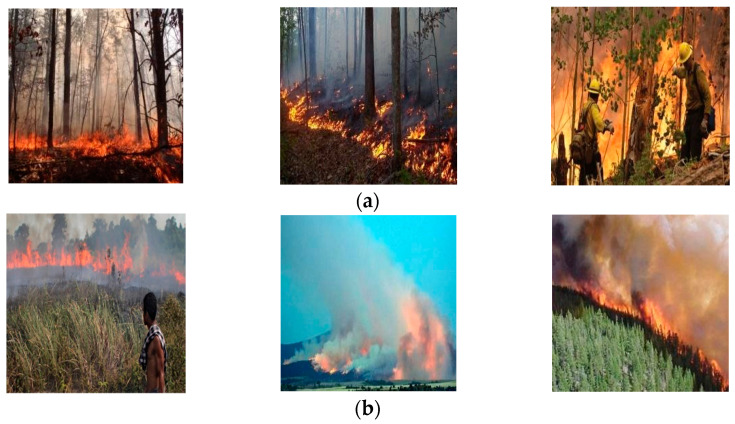
Fire images with different shapes (**a**) close by and (**b**) far away. (**a**) Close-distance fire images. (**b**) Long-distance fire images [6].

False detection in real-time is inconvenient. After detecting these errors, we upgraded our experiment using new training parameters. Thus, we realized that the mask-RCNN model was more accurate than improving our parameters. Fire has no specific shape and color and has different hues, saturation, and exposure as shown in Figure 6. Therefore, during training, changing those parameters randomly provides better results.

We changed our approach to our dataset owing to false image detection of hue and opacity. In our dataset, there were low-quality images with sizes smaller than 512 × 512. Therefore, we decided not to use automatic hue, exposure, or saturation values. Moreover, before training our model, we increased our dataset using an algorithm depending on the pixel value, brightness, and contrast value, and the example of the pixel transformation is as follows:

*Ց* (*x*) = *α*
*f(x)* + *β*     (*α* > 0) (1)

**Figure 7:**
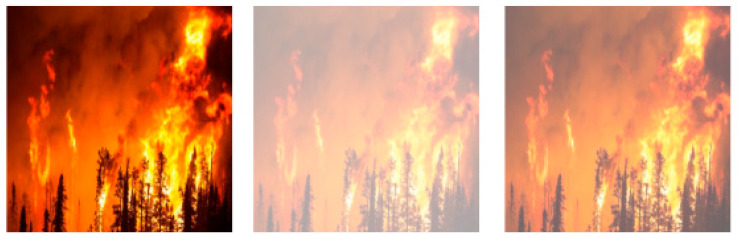
Fire images before and after hue augmentation.

**Scheme 3:**
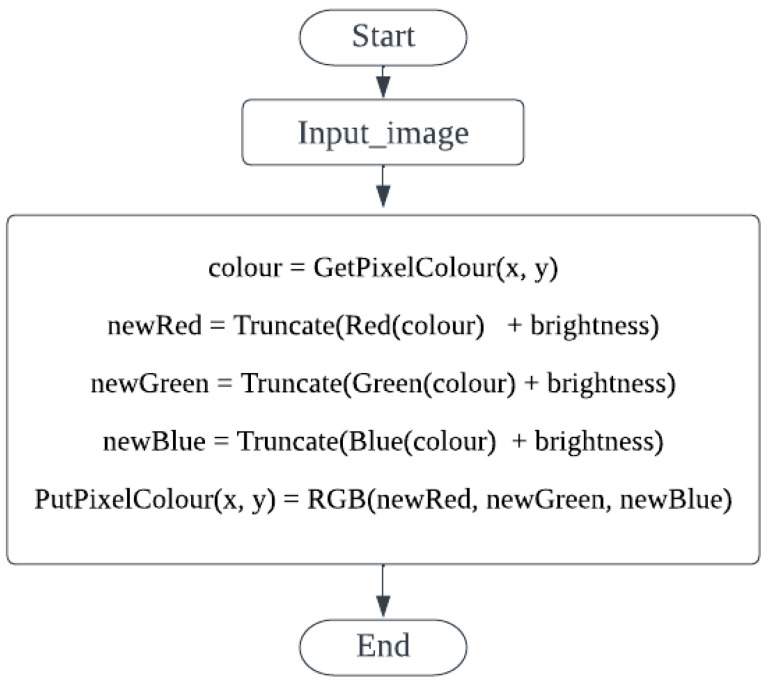
Image brightness.

In Equation (1), the parameters α > 0 and *β* are often called the gain and bias parameters. Here, these parameters are called to control contrast and brightness, respectively. *f*(*x*) refers to the source image pixels, and *g*(*x*) is the output image pixels. Then, more conveniently we can write the expression as Equation (2):

*ց* (*i*, *j*) = *α*
*f*(*i*, *j*) + *β* (2)

where *i* and *j* refer to the pixel locations in the *i*-th row and *j*-th column, respectively. The contrast value differs by changing the value of *α* from 1.0 to 3.0, and *β* refers to a brightness value of 0 to 100. Using this formula, we can change the contrast and brightness of the new data in our database, as shown in Figure 7. Scheme 3 shows the flow chart of image brightness. Here, PutPixelColour(x, y) is the representation of *ց* (*i*, *j*) function, and the color image with three-channel parameter values is changed by using three variables: newRed, newGreen, and newBlue.

**Scheme 4:**
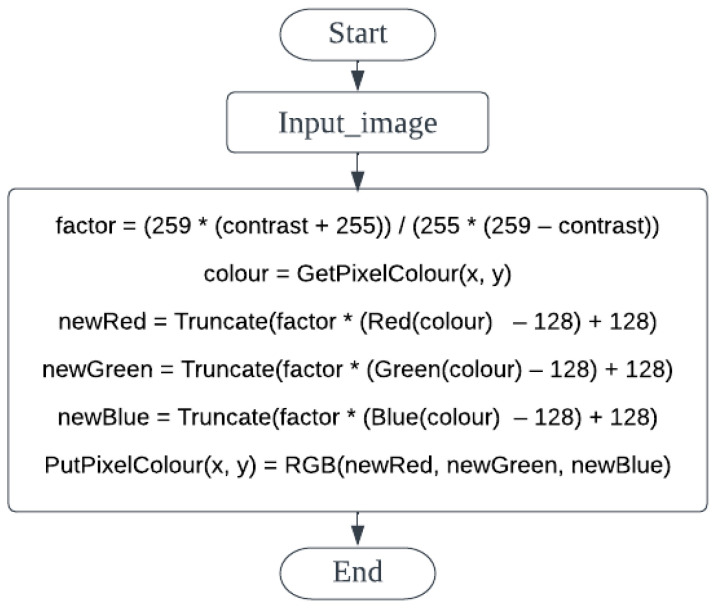
Image contrast.

Scheme 4 shows the flow chart of image contrast. Here, PutPixelColour(x, y) is the representation of the *ց* (*i*, *j*) function, and the color image with the three-channel parameter value is changed by using three variables: newRed, newGreen, and newBlue. The factor variable stores the main algorithm.

As mentioned in Section 3.1, our dataset contained 109,480 forest fires and 10,120 non-fire images. After a custom analysis of our database, we deleted low-quality and low-resolution images and obtained 116,200 images. After using the formula and algorithm for contrast and brightness of fire images, our dataset size increased to 348,600 from 119,600 images, as shown in Table 4. First, in our dataset, we doubled the contrast and reduced the brightness by half compared with the original images.

In the next subsection, we train our Detectron2 model using the same dataset and images of the same size. However, we observed that our training model provided significantly better results than before. The accuracies achieved are summarized in Table 5.

Using our new dataset, which has 348,600 images, we trained our model, as shown in Table 5. According to Table 5, Mask_rccn_50_FPN_3x had a high training accuracy of 98.3% and a testing accuracy of 97.8%, followed Keypoint_rcnn_R_50_FPN_3x, with 96.1% training accuracy and 95.3% testing accuracy, with a difference of less than 2%. Panoptic_fpn_R_101_3x also improved the training and testing accuracies by 88.3% and 85.1%, respectively. However, all models demonstrated increased training times compared to the last proposed model because of the large dataset.

**Figure 8:**
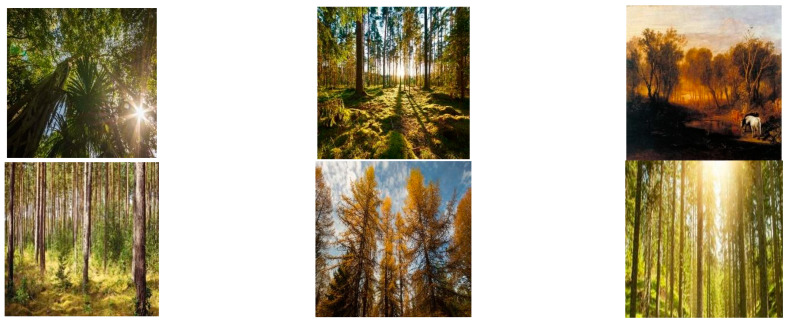
Examples of sunlight with different weather and season images in the dataset.

To achieve better accuracy in real-time, we also included 13,800 non-fire images, similar to the fire images. As previously mentioned, non-fire images achieve better real-time forest fire detection, which reduces false alarms. In general, sunlight is the most destructive method for the real-time detection of forest fires. Because of this, our large dataset will allow us to distinguish sunlight under different forest weather conditions, as shown in Figure 8 for sunrise and sunset during the day.

**Figure 9:**
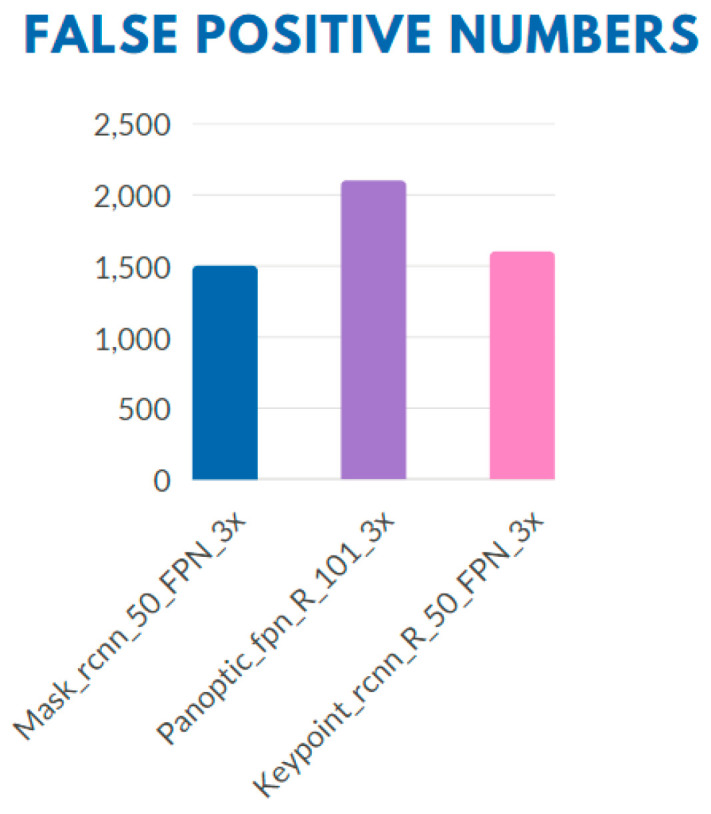
Result of the first experiment weight file for the false-positive tests.

We tested our different algorithms, and, as shown in Figure 9, Mask_rcnn_50_FPN_3x scored the lowest. In contrast, Panoptic_fpn_R_101_3x scored the highest.

**Figure 10:**
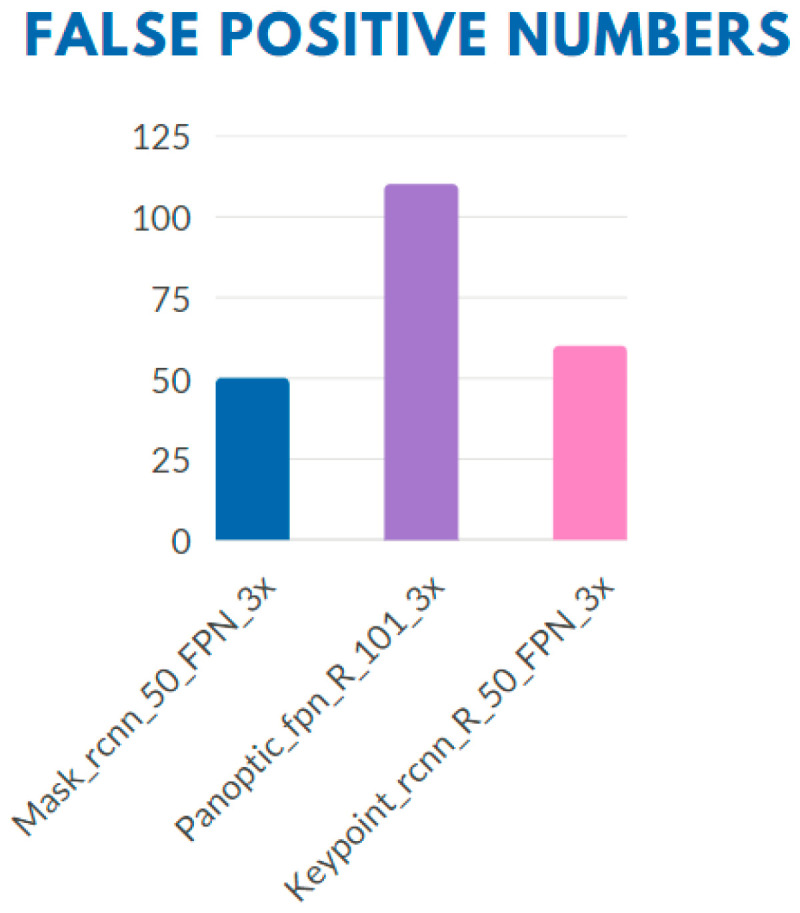
Results of the second experiment weight file for the false-positive tests.

According to Figure 10, our model showed a more positive output. After adding non-fire images to our dataset, our model dramatically improved, as shown.

**Figure 11:**
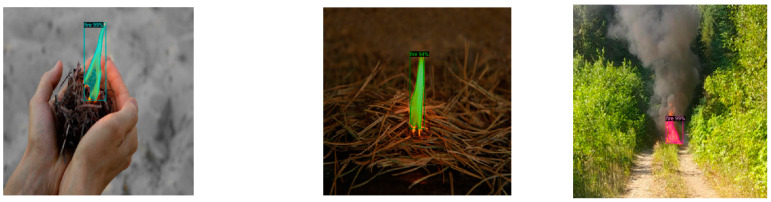
Small-size fire detection.

We achieved a maximum of 98.3% accuracy with our model. However, our approach failed to detect small forest fires. To achieve better accuracy, we included small images in our dataset to improve our final model, as shown in Figure 11. We employed a large-scale feature map to detect small moving objects and concatenated them with a feature map from earlier layers, which helped to preserve the fine-grained feature, as mentioned in [44]. This large-scale feature map with the location information of the previous layers and complex features of deeper layers was applied to identify small-sized fire pixels.

**Figure 12:**
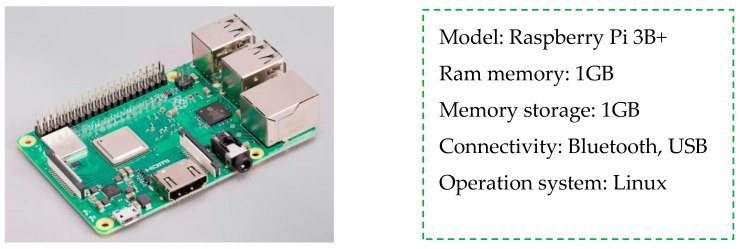
Characteristics of Raspberry Pi 3B+.

After the final training, our model accuracy increased to 99.3%, and it was possible to detect the size and color of the forest fires. Finally, we implemented our Mask _rcnn_50_FPN_3x model in Raspberry PI 3B+, as shown in Figure 12. The proposed method can be used for different CNNs. However, it responds faster in a small CNN than in a large CNN model. Our model achieved 99.3% accuracy performance, and compared with other state-of-the-art approaches, this model had fewer fire pixel misclassifications.

In Table 6, we compare our proposed model with an existing model to analyze its performance. An explanation is shown in the Section 4.

## 4. Experimental Results and Discussion

### Test with Fire and Non-Fire Images

**Figure 13:**
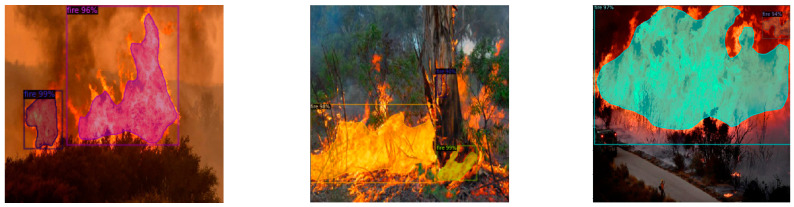
Results of forest fire detection for day images.

**Figure 14:**
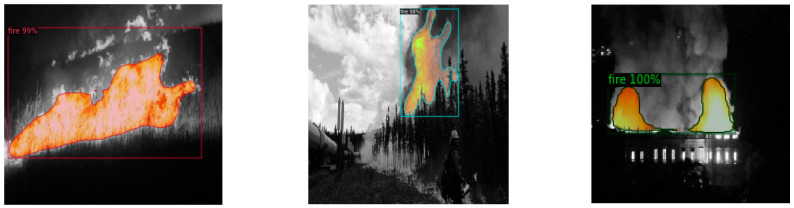
Results of forest fire detection for night images.

We implemented and tested our model using Visual Studio 2022 C++ on our laptop with a CPU speed of 3.20 Hz, 32 GB RAM, and 3GPU. To test our forest fire detection model, we implemented it in different environments. In previous sections, we discussed and implemented our model using Detectron2. This section discusses the strengths and limitations of the proposed model. Traditionally, the Faster-RCNN framework has been used to detect real-time fire, and its accuracy is quite high. However, our proposed model improved fire detection more than traditional forest fire detection methods and showed that the mask RCNN can achieve an accuracy of 99.3%. To achieve high accuracy, our model was trained with different parameters: hue, saturation, opacity, and small image pixels. In addition, the proposed model worked effectively under different circumstances, as shown in Figure 13 and Figure 14.

In this subsection, we discuss the compression of our model using different parameters and approaches. We used Detectron2 deep learning with a custom dataset to accurately detect forest fires with our model. In preparation for our study, we analyzed previous approaches. However, owing to the limitation of excess source code being publicly available and true object detection collaboration when initializing our model, as we mentioned earlier, our approach used a three-layer upgrade to reach the highest accuracy of 99.3% in our model. We tested the F-measure (FM), which measures the weighted average and balances the precision and recall rates. This score considers the false-negative and true-positive rates. Because measuring the accuracy rate is difficult, the FM is the most commonly used parameter to detect an object. In a detection model using the same weight, false negatives and true positives were better. However, if true positives and false negatives are dissimilar, precision and recall must be considered. Precision is the ratio of true positive observations.

In contrast, recall is a false-positive observation ratio, as detailed in previous research [45,46,47,48,49,50,51,52]. The precision of our proposed model was 99.3%, and the false detection rate was 0.7%. The following equations can be used to calculate the average precision and recall rates of our proposed method.

(3) $$Precision=\frac{TP}{TP+FP}$$

(4) $$Recall=\frac{TP}{TP+FN}$$

where *TP* refers to the true positive correctly detecting a forest fire and *FP* refers to false-negative detection regions. The relationship between precision and recall using the *FM* is shown in Equation (5):

(5) $$FM=\frac{2\times precision\times recall}{precision+recall}$$

Depending on the weather, reflection, darkness, and sunlight, actual forest fire images can be darker and blurred. Table 7 compares the recently published fire detection methods with the proposed method.

In Table 8, we compare our proposed model with other models according to different criteria. Based on the comparison, it is evident that our model does not suffer from extreme environments, such as dark, rainy, or sunny days, because of the inclusion of different sizes and contrast images for training. In addition, our model is more accurate under extreme weather conditions than other methods (Table 8).

The results of the model differ depending on different types of classifications as powerful, normal, and not strong (weak) among the seven aforementioned criteria. Powerful implies that the algorithm can be implemented for all kinds of events, and normal means the algorithm can fail in sudden cases. However, neither strong nor weak implies that the algorithm fails frequently based on color, opacity, image noise, and even size.

## 5. Limitations

**Figure 15:**
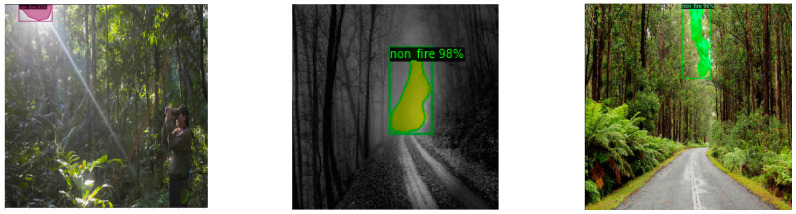
Limitation results for day and night non-fire images.

As mentioned in Table 8, a good or bad model cannot be determined based on specific criteria other than overall performance. Our proposed model has some limitations; for example, electric light or sun was considered fire in some cases when we tested the model in different environments, as shown in Figure 15. We intend to upgrade the proposed model using more datasets from different environments to solve this problem [57,58,59]. Furthermore, we did not create any classes for smoke in the custom dataset. Therefore, in the initial fire stage, if only smoke is present, our model waits until it detects a fire. As aforementioned, we are working on improving our model to overcome the aforementioned issue employing very large-scale datasets such as JFT-300M [60,61], which contains 300 million labelled images.

## 6. Conclusions

Numerous studies have been conducted to improve forest-fire detection systems using CNN-based deep-learning models. However, the Detectron2 deep learning model has not been explored for its potential in forest fire detection. Collecting sufficient image data for training models in forest fire detection is challenging, leading to data imbalance or overfitting concerns that impair the model’s effectiveness. In this study, we proposed a method to detect forest fires using the improved Detectron2 model and created a dataset.

First, we detected forest fires with a model to detect fires and subsequently with a different deep-learning object detection model. Next, we prepared our dataset, and to detect fire more accurately in the different stages and scenarios, we upgraded the dataset with small images and deleted low-quality pixel images. In addition, to expand our dataset, we used data augmentation algorithms to create 23 times more varied images from the original image. We experimentally compared the proposed method with existing methods to verify the model’s accuracy. After achieving the highest accuracy, we implemented our model in Raspberry Pi 3B+, which allowed us to run both the CPU and GPU details.

Furthermore, we observed some limitations in real-time applications, such as not labeling smoke images from our dataset. Future tasks include solving blurry problems under dark conditions and increasing the accuracy of the approach. We plan to develop a small model with reliable fire detection performance using 3D CNN/U-Net in the recognition and healthcare environments [62,63,64,65,66,67,68].

## Figures and Tables

**Table 1:** The custom dataset of the forest fires.

Dataset	Google, Bing, Kaggle, Flickr Images	Video Frames	Total
Forest Fire Images	2336	2864	5200

**Table 2:** Distribution of fire and non-fire images in the dataset.

Dataset	Training Images	Testing Images	Total
Fire Images	119,600	3300	122,900
Non-fire Images	10,120	0	10,120

**Table 3:** Pre-trained weights were obtained using a limited dataset.

Models	Input Size	Training Accuracy (AP50)	Testing Accuracy (AP50)	Weight Size	Iteration Number	Training Time
Mask_rcnn_50_FPN_3x	512 × 512	83.8%	79.8%	186 MB	50,000	62 h
Panoptic_fpn_R_101_3x		79.1%	76.9%	77 MB		47 h
Keypoint_rcnn_R_50_FPN_3x		82.4%	77.8%	152 MB		53 h

**Table 4:** Distribution of all fire images in the dataset.

Before	After Filtering	After Contrast Increased (Double)	After Contrast Decreased (Half)
119,600	116,200	116,200	116,200

**Table 5:** Pre-trained weights using Detectron2.

Algorithm	Input Size	Training Accuracy (AP50)	Testing Accuracy (AP50)	Weight Size	Iteration Number	Training Time
Mask_rcnn_50_FPN_3x	512 × 512	98.3%	97.8%	186 MB	50,000	89 h
Panoptic_fpn_R_101_3x		88.3%	85.1%	77 MB		71 h
Keypoint_rcnn_R_50_FPN_3x		96.1%	95.3%	152 MB		97 h

**Table 6:** Comparison between different models.

Features	R-CNN	Faster R-CNN	Our Method (Detectron2)
Test speed/s	49 s	2.3 s	Faster than 2 s
Real-time implementation	Not possible	Possible	Possible
Small object detection	Possible (accuracy less than that of Faster R-CNN	Possible (accuracy less than that of Mask R-CNN	Possible (highly accurate)
Algorithm	Selective search	Selective search	segmentation

**Table 7:** Quantitative results of fire detection.

Algorithm	P (%)	R (%)	FM (%)	Average (%)
Dilated CNNs [7]	98.9	97.4	98.2	98.1
AlexNet [53]	73.3	61.3	75.1	79.9
Faster R-CNN [54]	81.7	94.5	87.2	97.8
ResNet [55]	94.8	93.6	94.2	94.3
VGG16 [56]	97.5	87.9	92.7	92.6
Our Method (Detectron2)	99.3	99.4	99.5	98.9

**Table 8:** Forest fire detection performance comparison using various features.

Criterion	Dilated CNNs [6]	Faster R-CNN [14]	Our Method (Detectron2)
Scene independence	powerful	normal	powerful
Object independence	powerful	powerful	normal
Fire independence	powerful	normal	powerful
Robust to color	normal	not strong	powerful
Robust to noise	normal	powerful	powerful
Fire-spread detection	no strong	not strong	powerful
Computational load	powerful	normal	powerful

## Data Availability

Not applicable.
